# GWLD: an R package for genome-wide linkage disequilibrium analysis

**DOI:** 10.1093/g3journal/jkad154

**Published:** 2023-07-11

**Authors:** Rong Zhang, Huaxuan Wu, Yasai Li, Zehang Huang, Zongjun Yin, Cai-Xia Yang, Zhi-Qiang Du

**Affiliations:** College of Animal Science, Yangtze University, Jingzhou 434025, Hubei, China; College of Animal Science, Yangtze University, Jingzhou 434025, Hubei, China; College of Animal Science, Yangtze University, Jingzhou 434025, Hubei, China; College of Animal Science, Yangtze University, Jingzhou 434025, Hubei, China; College of Animal Science and Technology, Anhui Agricultural University, Hefei 230036, Anhui, China; College of Animal Science, Yangtze University, Jingzhou 434025, Hubei, China; College of Animal Science, Yangtze University, Jingzhou 434025, Hubei, China

**Keywords:** linkage disequilibrium, mutual information, reduced mutual information, genome-wide analysis, Rcpp package, C++

## Abstract

Linkage disequilibrium (LD) analysis is fundamental to the investigation of the genetic architecture of complex traits (e.g. human disease, animal and plant breeding) and population structure and evolution dynamics. However, until now, studies primarily focus on LD status between genetic variants located on the same chromosome. Moreover, genome (re)sequencing produces unprecedented numbers of genetic variants, and fast LD computation becomes a challenge. Here, we have developed GWLD, a parallelized and generalized tool designed for the rapid genome-wide calculation of LD values, including conventional *D*/*D*′, $r^{2}$, and (reduced) mutual information (MI and RMI) measures. LD between genetic variants within and across chromosomes can be rapidly computed and visualized in either an R package or a standalone C++ software package. To evaluate the accuracy and speed of LD calculation, we conducted comparisons using 4 real datasets. Interchromosomal LD patterns observed potentially reflect levels of selection intensity across different species. Both versions of GWLD, the R package (https://github.com/Rong-Zh/GWLD/tree/master/GWLD-R) and the standalone C++ software (https://github.com/Rong-Zh/GWLD/tree/master/GWLD-C%2B%2B), are freely available on GitHub.

## Teaser

Until now, LD analysis has focused on genetic variants on the same chromosome. Additionally, the large number of variants produced by current sequencing technologies challenges LD computing speed. Zhang *et al*. present GWLD: a parallelized and generalized tool for rapid, genome-wide calculation and visualization of LD values. The authors compare the accuracy and speed of LD calculation between GWLD and other software packages and find that interchromosomal LD patterns from different species potentially reflect the level of selection intensity.

## Introduction

Linkage disequilibrium (LD) refers to the nonrandom association of alleles between 2 genetic loci. LD analysis is an important tool for population genetics and evolution and has been widely applied in dissecting the genetic architecture of complex traits, such as human disease and animal and plant breeding (Sved and Hill 2018). A number of tools have been developed to expedite LD calculation (Quick *et al.* 2019; Myers *et al.* 2020; Tang *et al.* 2020; Theodoris *et al.* 2021; Lin *et al.* 2022), to enhance LD pattern visualization (Luna and Nicodemus 2007; Kim *et al.* 2019; He *et al.* 2020; Dong *et al.* 2021; Privé 2021), and to facilitate the annotation of genetic variants (Prunier *et al.* 2019; Zhang *et al.* 2019; Mansour *et al.* 2021). However, until now, LD status has evaluated only genetic variants on the same chromosome. High-order LD was suggested to be 1 possible explanation for statistical epistasis, as revealed in the genome-wide analysis (Ella *et al.* 2010; Zan *et al.* 2018; Domingo *et al.* 2019). Therefore, the evaluation of interchromosomal LD patterns could provide novel insights for complex trait genetics research.

With the advancement and application of next-generation sequencing, genetic variants can now be produced at an unprecedented level. Efficient genome-wide LD calculation is crucial to save time and resources, so the development of new methods or software packages is needed. Compared with traditional LD measures (*D*/*D*′ and $r^{2}$), the mutual information (MI) and reduced mutual information (RMI) can be considered LD measures, which calculate information shared by 2 variables (Newman *et al.* 2020), and have been used for multilocus haplotype LD (Okada 2018), tag SNP prioritization (Liao *et al.* 2017), and epistasis analyses (Heinrich *et al.* 2021).

Here, we have developed GWLD, an R package designed to calculate and visualize genome-wide LD values (*D*/*D*′, $r^{2}$, MI, and RMI). The LD measures were computed (in a parallelized and high-speed fashion), visualized (in the traditional heatmap and circos-like map modes), and further analyzed based on R tools (R Core Team 2022). We analyzed 4 real datasets (duck, human, pig, and maize) by GWLD, with the scale of numbers of genetic variants and samples ranging from small to large. To enhance computational efficiency, a standalone C++ software package was also developed. Additionally, the computational efficiency and power between GWLD and other tools (R packages: Genetics, Infotheo; software: Tassel, Plink) were compared.

## Materials and methods

### LD measures

The conventional LD measures, D and $r^{2}$, are computed between 2 genetic loci A and B (biallelic), as defined in quantitative genetics (Sved and Hill 2018):


(1) $$D=P_{A1B1}-P_{A1}\;*\;P_{B1}$$

(2) $$r^{2}=\frac{D^{2}}{P_{A1}(1-P_{A1})P_{B1}(1-P_{B1})}$$

*P*_A1_, *P*_B1_, and *P*_A1B1_ refer to the allele and haplotype frequencies. For diploid individuals, however, direct inference of haplotype frequencies is usually impossible when the gametic phase is unknown. The reason is that the exact haplotype configurations for double heterozygotes cannot be discerned, as they can be either *AB*/*ab* or *Ab*/*aB*. Under Hardy–Weinberg equilibrium, the expected frequencies for each genotype *f*_11_, *f*_12_, …, *f*_33_ are shown in Table 1, and the log-likelihood with respect to the haplotype frequencies is


(3) $$log\;L(P_{AB},P_{Ab},P_{aB},P_{ab})=\overset{3}{\underset{i,j=1}{\sum }}\;n_{ij}\;*\;log(\;f_{ij})+\;constant$$

**Table 1.** Observed genotype numbers and expected frequencies between 2 diallelic loci.

	*BB*	*Bb*	*bb*
*AA*	$n_{11},\;f_{11}=f_{AB}f_{AB}$	$n_{12},\;f_{12}=2f_{AB}f_{Ab}$	$n_{13},f_{13}=f_{Ab}f_{Ab}$
*Aa*	$\;n_{21},f_{21}=2f_{AB}f_{aB}$	$\;n_{22},f_{22}=2f_{AB}f_{ab}+2f_{aB}f_{Ab}$	$n_{13},f_{23}=2f_{Ab}f_{ab}$
*aa*	$\;n_{31},f_{31}=f_{aB}f_{aB}$	$n_{32},\;f_{32}=2f_{aB}f_{ab}$	$n_{13},f_{33}=f_{ab}f_{ab}$

In information theory, entropy and MI are 2 different basic concepts. Entropy quantifies the uncertainty provided by a random variable.


(4) $$H(X)=-\underset{x}{\sum }\;p(x)log(\;p(x))$$

However, MI calculates the interdependence between 2 random variables.


(5) $$\begin{array}{l} MI(r;s)=H(s)-H(s|r)\\ \;\;\;\;\;\;=\;-\underset{s}{\sum }\;p(s)log\;p(s)+\underset{r,s}{\sum }\;p(r,s)log\frac{\;p(r,s)}{\;p(s)}\\ \;\;\;\;\;\;=\underset{r,s}{\sum }\;p(r,s)log\left(\frac{\;p(r,\;s)}{\;p(r)p(s)}\right) \end{array}$$

The RMI was proposed recently to quantify more precisely the between-variable relationship (Newman *et al.* 2020).


(6) $$RMI=\frac{1}{n}\left[log\frac{n!\prod_{rs}\;c_{rs}!}{\prod_{r}\;a_{r}!\prod_{s}\;b_{s}!}-log\;\Omega (a,b)\right]$$

In which, $\frac{1}{n}\log\frac{n!\prod_{rs}\;c_{rs}!}{\prod_{r}\;a_{r}!\prod_{s}\;b_{s}!}≅\sum_{r,s}\;p(r,s)log\frac{\;p(r,s)}{\;p(r)p(s)}$.

Here, MI and RMI were employed to calculate the relationship between 2 genetic variants.

### Datasets

Four datasets were used for software performance evaluation and benchmark analysis, as compared with Tassel (Bradbury *et al.* 2007), Plink (Purcell *et al.* 2007), R Genetics package (for $r^{2}$ comparison), and R Infotheo package (for MI comparison). Quality control was performed by Plink with the following parameters (duck data, –geno 0.1, –mind 0.1, –maf 0.05, –hwe 0.001; pig and human data, –geno 0.1, –mind 0.1, –maf 0.05, –hwe 0.001, –ld-window-kb 1000, –ld-window-r2 0.2; and maize data, –geno 0.1, –mind 0.1, –maf 0.05).

After quality control, the duck dataset contained 40,601 single nucleotide polymorphisms (SNPs) across 29 chromosomes for 542 Pekin ducks (Deng *et al.* 2019). The pig data contained 44,498 SNPs for 2,549 animals (Tan *et al.* 2017; Yang *et al.* 2021). The human data were relatively large, containing 250,486 SNPs for 2,504 individuals (Fairley *et al.* 2020). The maize data were composed of 43,090 markers for 270 inbred line samples (Cook *et al.* 2012).

### GWLD overview

GWLD quickly generated and plotted LD results on large-scale genetic variants data at the genome-wide level, specifically designed for markers on different chromosomes. Genotype data in either variant call format (VCF) or Plink file format were used as inputs for calculating LD measures (*D*/*D*′, $r^{2}$, MI, and RMI). Outputs were exported as plots (heatmap, LD decay, and circos-like), txt files, or other formats as desired.

The R package (GWLD-R) was developed in the R environment with the Rcpp package, which was fast in speed and capable of handling huge volumes of data inputs. Additionally, a standalone software version, GWLD-C++, was implemented in C++. Moreover, the parallel computing option was added.

**Fig. 1.**
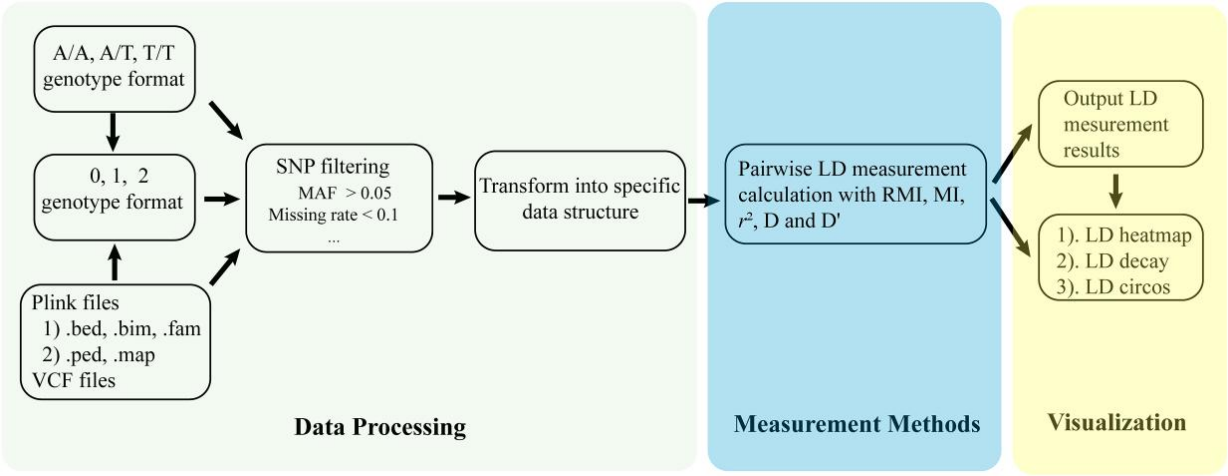
GWLD overview. Mainly composed of 3 modules: data processing, computation of LD measures, and visualization.

GWLD was executed according to the following 3 steps: file format conversion and input, LD calculation, and visualization of LD results (Fig. 1, Box 1). GWLD had full documentation and user guides and was also under active development (Supplementary File 1).

**Fig. 2.**
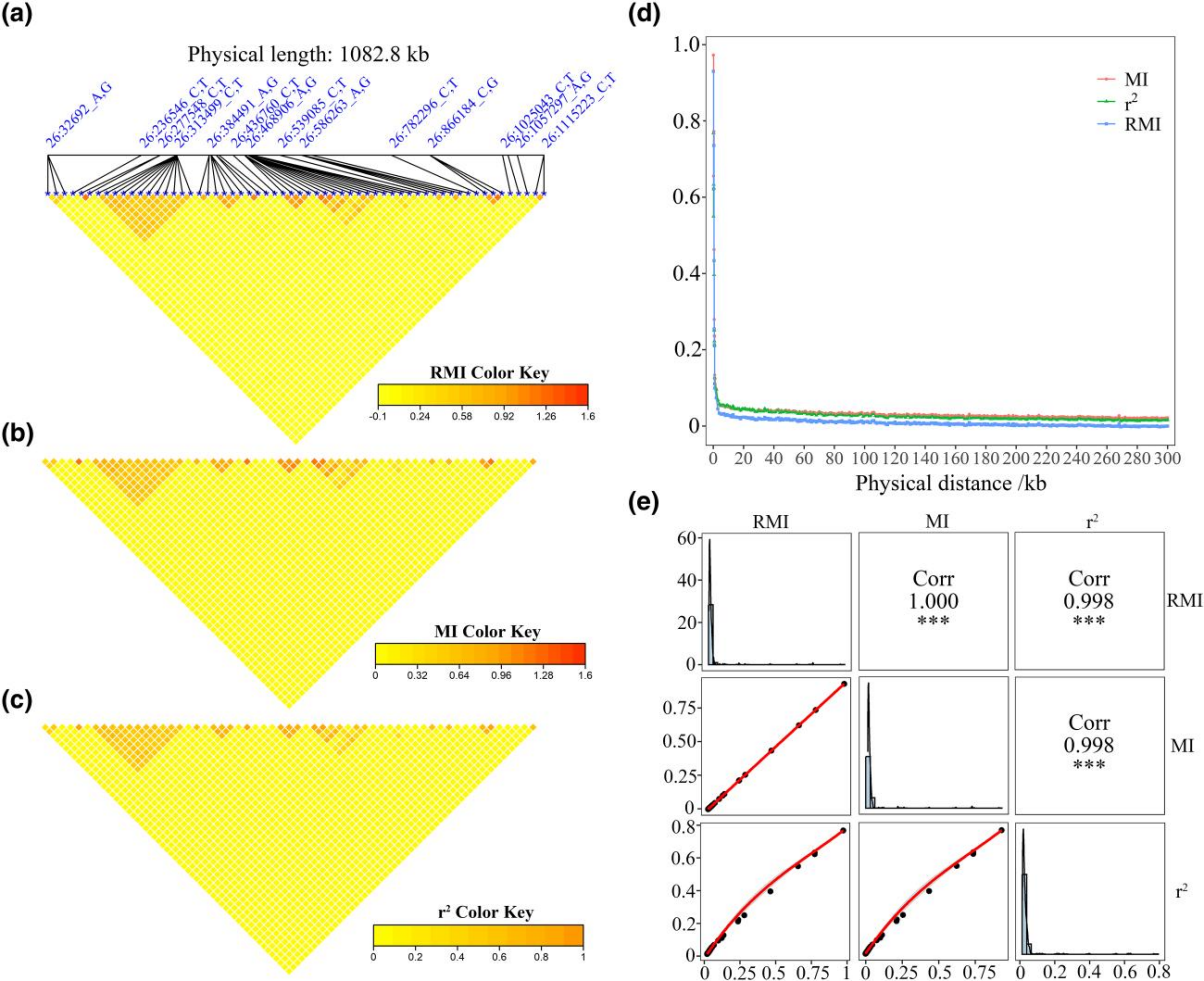
Correlation plots between LD measures. a–c) RMI, MI, and *r*^2^ heatmaps for duck chromosome 26. d) Genome-wide LD decay for ducks. e) Main diagonal, histogram and fitted curves of LD value distributions. Upper right, correlation between different LD measures. Lower left, scattered plots and fitted lines for different LD measures.

**Fig. 3.**
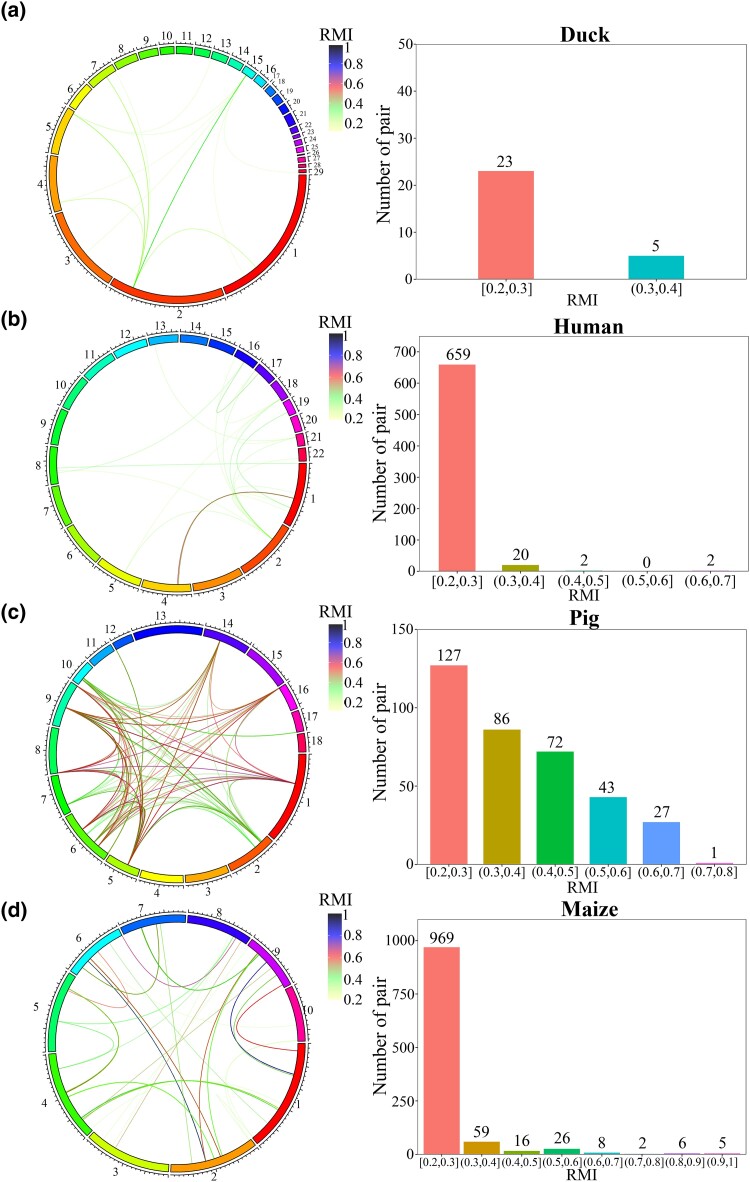
Genome-wide view of interchromosomal LD patterns. RMI was calculated and used as the LD measure. Pig and maize had more SNP pairs with high interchromosomal LD values. a–d) Duck, human, pig, and maize. Left panel, circos-like plots for interchromosomal LD values. Right panel, histogram of numbers of SNP pairs with different interchromosomal LD values. *x*-axis, distribution of LD intervals; *y*-axis, number of SNP pairs.

Box 1. **R script to demonstrate the calculation of genome-wide LD values using GWLD. The packages use the parallel computing mode, and different LD measures can be computed (Fig. 2); visualization of LD decay (Fig. 2) and circos-like plots (Fig. 3) can also be done.**

##install the GWLD packages

devtools::install_github(‘Rong-Zh/GWLD/GWLD-R’)

##using packages

library(GWLD)

#0) Loading example data with base-type genotype

data(duck)

data <- duck$SNP

SNP <- data$genotype

Info <- data$info

## Read data from vcf or plink format file

# Recode the genotypes in vcf file as 0, 1, 2, NA, or recode to another type

vcf_data <- read.vcf(“vcf format file”, genotype=“int”)

plink_data <- read.plink(“plink format file’s prefix”)

#1) recode the genotype with 0,1,2,NA(NA for missing values)

SNP <- codegeno(SNP, sep=“/”)

#2) calculate with different methods (D, D’, r2, RMI, MI)

result <- GWLD(SNP, method = “r^2”, cores = 1)

##or use the following code `

r2 <- LD(SNP, method = “r^2”, cores = 1)

rmi <- RMI(SNP, cores=1)

mi <- MI(SNP, cores=1)

#2.1) LD heatmap plot

p <- HeatMap(SNP, method = “RMI”, SnpPosition = Info$POS, SnpName = Info$ID, cores = 1, color = “YellowTored”, showLDvalues = F)

#3) decay from result(step 2)

rmi_decay <- decay(rmi, Info)

#or calculate circos from data

rmi_decay <- calc_decay(SNP, Info, method=“RMI”)

#4) circos from result(step 2)

rmi_circos <- circos(rmi, Info, threshold=0.2)

#or calculate circos from data

rmi_circos <- calc_circos(SNP, Info, method=“RMI”, threshold=0.2)

#4.1) circos plot

circosdata <- duck$Circos

circos.ideogram(circosdata$chr)

circos.linksnp(circosdata$linkdata, bg.col=rainbow(29))

#### Step 1: genotype data processing

The input file for GWLD can be prepared in either VCF or Plink format (i.e. files with suffixes, bed, fam, and bim or ped and map). Genotype data can be arranged as raw nucleotides or recoded as 0, 1, and 2 (representing genotypes, e.g. AA, AG, GG, or in other formats).

#### Step 2: LD calculation

Once the input files are read into the software and transformed into a genotype matrix, GWLD allows for the calculation of LD measures such as *D*/*D*′, $r^{2}$, MI, and RMI. Moreover, the parallel computing option can be selected to accelerate the computation process (argument, the number of cores, can be set as 1 or many, i.e. the usage of single or multiple threads).

#### Step 3: LD pattern visualization

The calculated LD results can be visualized either as LD heatmap and LD decay plots (for all measures, *D*/*D*′, $r^{2}$, MI, and RMI) or as circos-like circular genome-wide LD plots (for MI and RMI only). Output results can be exported as txt files (by chromosome and between pair of chromosomes), and plots can also be exported as figures in proper suffixes.

## Results and discussion

### GWLD demonstration

First, we evaluated the consistency of conventional LD values (*D*/*D*′ and $r^{2}$) computed by GWLD and the R Genetics package and Tassel and Plink software. Next, MI values were compared between those calculated by GWLD and the R Infotheo package. Perfect matches of these different LD values were found.

Moreover, for all 4 datasets, high Pearson’s correlations (on average >0.95) existed among MI, RMI, and $r^{2}$ values (Supplementary Tables 1–4). Furthermore, using the duck data as an example, MI, RMI, and $r^{2}$ values were shown to have highly similar heatmaps (duck chromosome 26) (Fig. 2a–c) and genome-wide decay patterns as well (Fig. 2d). Perfect Pearson’s correlations between MI and RMI were also found (Fig. 2e).

After interchromosomal LD analysis (RMI only), a circos-like genome-wide plot clearly showed that a nonrandom association of alleles between genetic loci on different chromosomes existed (Fig. 3). However, interestingly, for the 4 datasets, different levels of interchromosomal LD were observed, and the majority of variant pairs had LD values within the range of [0.2, 0.3]. For ducks, only 5 pairs of SNPs existed in the (0.3, 0.4] range (Fig. 3a), and for humans, 20 pairs were within (0.3, 0.4], and 2 pairs of SNPs were in each of the ranges (0.4, 0.5] and (0.6, 0.7], respectively (Fig. 3b). Notably, for pig and maize datasets, under intensive breeding and selection pressures, 28 and 21 pairs of SNPs distributed in the LD regions with values > 0.6, respectively (Fig. 3c and d). Especially for maize, 11 pairs of SNPs had LD values > 0.8 (Fig. 3d).

### Computing speed

We demonstrated and compared the computing speeds required for the calculation of different LD measures using the duck dataset (on Linux, 4X 10-cores Intel(R) Xeon(R) CPU E7-4820 v4 @2.00GHz; 1T RAM).

**Fig. 4.**
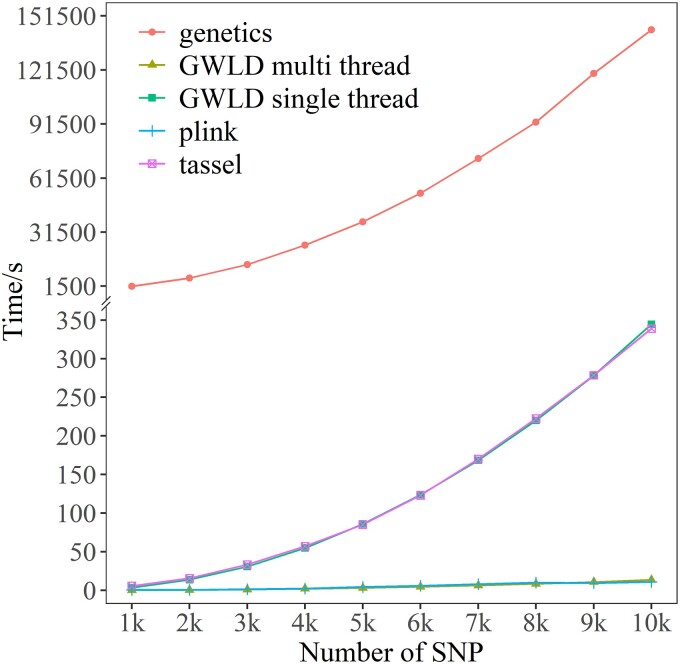
Benchmark analyses on computing speeds between GWLD and other software (Tassel, Plink, and R Genetics package). The duck data were used as an example.

First, benchmark analyses and comparisons between GWLD and different software and R packages were shown (Fig. 4). The R Genetics package was the slowest, and the computing speed dramatically decreased with the increase of SNP numbers. Tassel and GWLD single-thread versions performed with nearly the same speed, and the computing time increased about 7-fold (from 50 to 350 s, from 4,000 to 10,000 SNPs). However, Plink and GWLD multithread calculated with the fastest speed (perfect overlap with each other) and maintained consistent performance, not affected by the increase of SNP numbers.

Next, we showed that for 1,000 SNPs and 542 ducks, when choosing the single-thread mode, GWLD R-base scripts took 20.3 min for $r^{2}$ calculation but only 4.7 min for MI and 6.3 min for RMI. However, using scripts based on Rcpp packages, the speed was greatly accelerated, 1.56 s for $r^{2}$, 5.01 s for MI, and 5.36 s for RMI, respectively. In contrast, the Genetics package in R was slow and took 23.9 min for $r^{2}$ calculation.

**Fig. 5.**
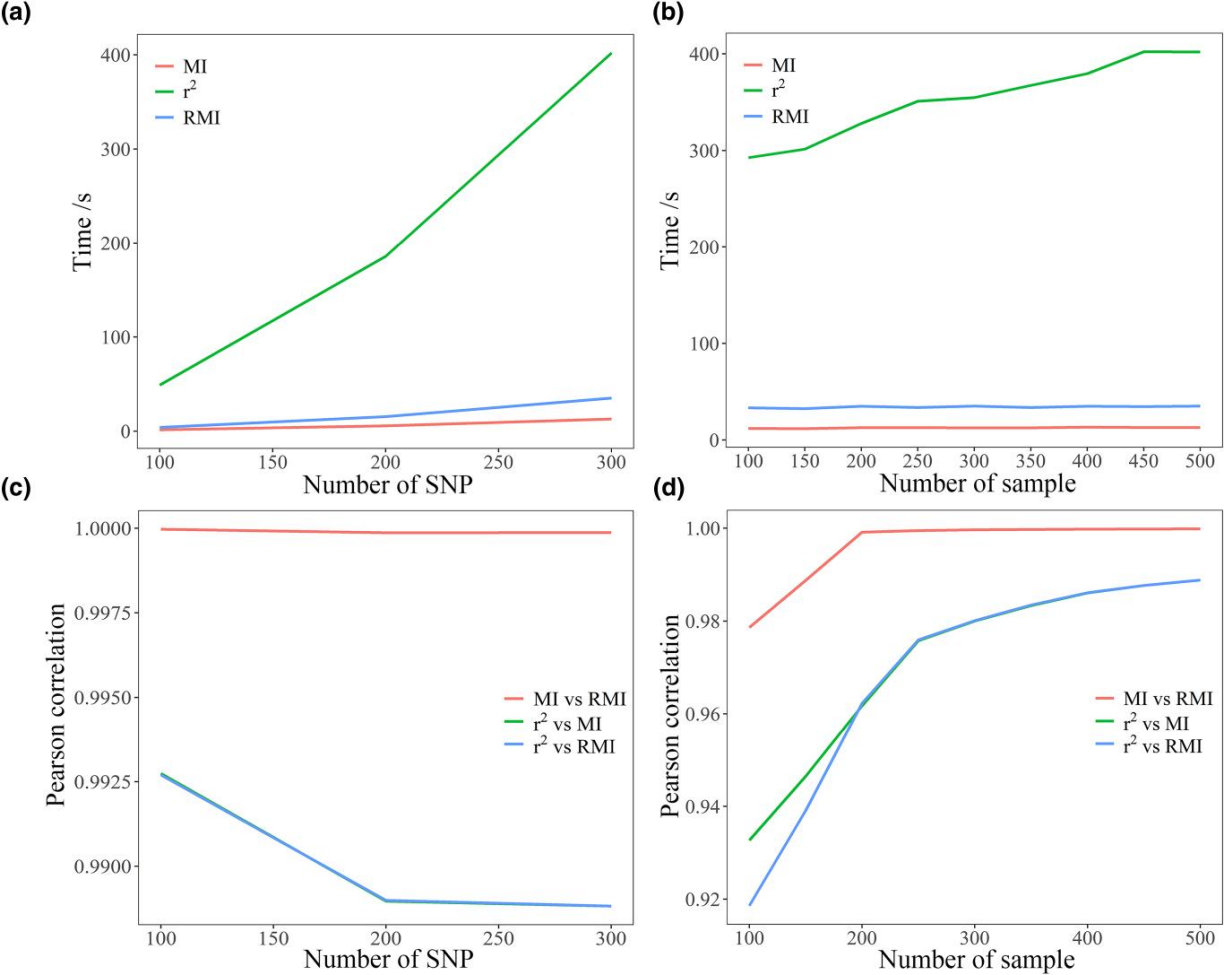
Evaluation of the effects of numbers of SNPs and samples on GWLD computing speeds. The duck data were used as an example. a and b) Time required for calculation (in seconds). c and d) Relative Pearson’s correlation between LD measures.

We further evaluated the impact of different numbers of SNPs and samples on computing speed. With the increase of SNP numbers and sample sizes, the amount of time was considerably increased, as required for computing $r^{2}$, nearly 8-fold (50–400 s) more, whereas few or nearly no impacts on calculating MI and RMI were found (Fig. 5a and b). Furthermore, generally highly consistent Pearson’s correlations (>0.98) between $r^{2}$, MI, and RMI were also found (Fig. 5c and d). Though with the increase of SNP numbers and sample sizes, slight decreasing and increasing patterns of correlations were found, respectively.

When using the complete duck dataset (∼40,000 SNPs, 542 samples), we found that for GWLD R-base scripts with the single-thread mode, calculation of $r^{2}$ needed >30 days (d), whereas for RMI and MI, 8.2 and 3.5 d, respectively. However, for the parallel mode (31 threads), the computation terminated much faster (>8-fold of less time needed) (3.7 d, 9.8 h, and 5.9 h, respectively) (Table 2). In contrast, when the Rcpp (RcppArmadillo + OpenMP) computation mode was selected (Table 2), the whole computing process was completed in hours (1.6, 3.7, and 4.0 h, for $r^{2}$, MI, and RMI), even for the single thread. With the multithread computing mode, less than half an hour (6.8, 18.4, and 25.3 min) was required.

**Table 2.** GWLD computing speed comparisons (the duck data).

	Method	Single Thread	Multithread (31)
R + parallel	RMI	8.2 d	9.8 h
	MI	3.5 d	5.9 h
	LD ($r^{2}$)	30+ d	3.7 d
RcppArmadillo + OpenMP	RMI	4.0 h	25.3 min
	MI	3.7 h	18.4 min
	LD ($r^{2}$)	1.6 h	6.8 min

Note: System configuration (Linux, 4X 10-cores Intel(R) Xeon(R) CPU E7-4820 v4 @2.00GHz; 1T RAM).

### Summary

Until recently, LD analyses still only consider the relationship of genetic variants on the same chromosome and focus on the incorporation of different formats of input data, the speed of calculation, and better visualization and annotation (Luna and Nicodemus 2007; Prunier *et al.* 2019; Quick *et al.* 2019; Zhang *et al.* 2019; He *et al.* 2020; Myers *et al.* 2020; Tang *et al.* 2020; Dong *et al.* 2021; Mansour *et al.* 2021; Theodoris *et al.* 2021). Here, we first showed that GWLD directly computes the MI between genetic variants and demonstrated the between-chromosome LD distribution and pattern. Computing speed has been greatly accelerated by C++ codes, and further optimization of the R packages and software design to accommodate more data computation is planned in the near future since currently it is not uncommon to face large volumes of genetics or genomics data due to the fast development and application of modern nucleic acid sequencing technology. In addition, the genome-wide LD distribution can be compared with genome-wide epistasis analysis to explore the genetic relatedness and interaction effects between genetic variants on complex traits of agricultural or medical importance (Ella *et al.* 2010; Zan *et al.* 2018; Domingo *et al.* 2019). As we found here that for species with a different selection or breeding history, a great diversity of interchromosomal LD patterns existed (e.g. pig and maize under strong selection had more numbers of SNP pairs showing high interchromosomal LD values). Estimation of MI can be further improved, not only through RMI but also through the Jackknife method, which can better capture the relationship between 2 random variables (Zeng *et al.* 2018; Hernández and Samengo 2019; Newman *et al.* 2020).

## Supplementary Material

jkad154_Supplementary_Data

## Data Availability

Four datasets can be accessed publicly: the duck (NCBI accession number: SRP155579), the pig (NCBI accession numbers: PRJNA681437 and PRJNA712489), the human (https://ftp-trace.ncbi.nih.gov/1000genomes/ftp/release/), and the maize (cbsusrv04.tc.cornell.edu/users/panzea/download.aspx?filegroupid=7). The GWLD R package and software source codes are available on GitHub (https://github.com/Rong-Zh/GWLD). Supplemental material available at G3 online.
