# Supplementary material for: GWLD: an R package for genome-wide linkage disequilibrium analysis
Source: G3 (Bethesda). 2023 Jul 11;13(9):jkad154. doi: 10.1093/g3journal/jkad154 (PMC10468308; doi:10.1093/g3journal/jkad154)
Supplement: jkad154_Supplementary_Data [file jkad154_supplementary_data.zip › File_S1_G3-2023-404081.docx]

# Manual for GWLD (an R package for genome-wide linkage disequilibrium analysis)

## Installation

1.1 Dependences

- Armadillo ( <http://arma.sourceforge.net/download.html>)
- Openmp

1.2 Cmake

- Install cmake >= 3.9 (<https://cmake.org/download/> )

Use the following commands to install GWLD:

- $ mkdir build && cd build
- $ cmake ..
- $ make
- $ ../bin/GWLD

1.3 compiling source codes

- $ g++ -std=c++11 -o bin/GWLD src/main.cpp src/filedeal.cpp src/methods.cpp src/utils.cpp -I include -larmadillo -fopenmp

## Arguments and parameters

| Short version | Long version | Parameter type | Description | Default |
| --- | --- | --- | --- | --- |
| -b | –bfile | string | Input plink format (*.bed *.bim and *.fam) file’s prefix | - |
| -v | –vcf | string | Input vcf format file name | - |
| -f | –file | string | Input plink format (*.ped and *.map) file’s prefix | - |
| -m | –method | string | Calculation method between two sites | RMI |
| - | –by-chrom | - | Calculated by chromosome | - |
| - | –decay | - | Output decay according to the chosen method | - |
| - | –max-dist | int | Calculates the maximum distance between two sites while decay parameter selected | - |
| - | –circos | - | Calculate between different chromosomes | - |
| - | –code012 | - | Recode genotype with -1, 0 ,1, 2. -1 for missing value | - |
| - | –allele-freq | - | Calculate allele frequency per site | - |
| - | –2vcf | - | Convert plink format file to vcf format file | - |
| -p | –threads | int | threads number | 1 |
| -o | –out-prefix | string | output file name’s prefix | gwld |
| -h | –help | - | print help message | - |

## Main function

To calculate the linkage disequilibrium (LD) measures, including D, D’, $r^{2}$, mutual information (MI) and reduced MI (RMI), the following options can be chosen.

### 3.1 Loci on the same chromosome (D, D’, $\boldsymbol{r}^{\boldsymbol{2}}$, MI and RMI)

./GWLD -vcf test.vcf -m RMI --by-chrom -p 10 -o result

### 3.2 Loci on two different chromosomes (MI and RMI)

./GWLD -vcf test.vcf -m RMI -p 10 -o result

### 3.3 LD decay at the global level (default, maximum distance 300kb between two loci, using the –max-dist parameter)

./GWLD -vcf test.vcf -m RMI -decay -p 10 -o result

### 3.4 Circos-like map visualization between different chromosomes

./GWLD -vcf test.vcf -m RMI -circos -p 10 -o result

## 4. Other functions

### 4.1 Exchange between different input file formats

Files in the plink format can be reformatted into variant calling format (VCF).

4.1.1 From Plink file formats (*.bed, *.bim and *.fam) to VCF

- ./GWLD -bfile test -2vcf -o result

4.1.2 Plink file formats (*.ped and *.map) to VCF

- ./GWLD -file test -m -2vcf -o result

### 4.2 Numbers, start and end positions of markers on each chromosome

./GWLD -vcf test.vcf --chr-info -o result

### 4.3 Allele frequency

./GWLD -vcf test.vcf --allele-freq -o result

### 4.4 Genotypes recoded into 0, 1, 2, -1 (missing values), and output as VCF files

4.4.1 Plink format (*.bed, *.bim和 *.fam)

- ./GWLD -bfile test --code012 -o result

4.4.2 Plink format (*.ped and *.map)

- ./GWLD -file test --code012 -o result

4.4.3 VCF

- ./GWLD -vcf test.vcf --code012 -o result
