# Supplementary material for: GWLD: an R package for genome-wide linkage disequilibrium analysis
Source: G3 (Bethesda). 2023 Jul 11;13(9):jkad154. doi: 10.1093/g3journal/jkad154 (PMC10468308; doi:10.1093/g3journal/jkad154)

**Table S1. Correlation between MI, RMI and r^2^ values on each chromosome (Duck)**

| Chromosome | Number of SNP | | RMI vs $r^{2}$ | MI vs$r^{2}$ | RMI vs MI |
| --- | --- | --- | --- | --- | --- |
| 1 | | 7692 | 0.881399 | 0.88312 | 0.995366 |
| 2 | | 6244 | 0.899551 | 0.900575 | 0.996035 |
| 3 | | 4451 | 0.917616 | 0.918608 | 0.998203 |
| 4 | | 2815 | 0.939936 | 0.940634 | 0.998009 |
| 5 | | 2722 | 0.942352 | 0.942749 | 0.999266 |
| 6 | | 1449 | 0.958281 | 0.958537 | 0.999409 |
| 7 | | 1669 | 0.953775 | 0.953924 | 0.999477 |
| 8 | | 1671 | 0.959249 | 0.95999 | 0.999043 |
| 9 | | 1353 | 0.966742 | 0.967113 | 0.999445 |
| 10 | | 717 | 0.981555 | 0.981693 | 0.999685 |
| 11 | | 660 | 0.973304 | 0.973655 | 0.999598 |
| 12 | | 749 | 0.971419 | 0.97136 | 0.999554 |
| 13 | | 1072 | 0.96757 | 0.967751 | 0.999538 |
| 14 | | 1049 | 0.971057 | 0.971374 | 0.999504 |
| 15 | | 706 | 0.970878 | 0.97111 | 0.999218 |
| 16 | | 639 | 0.981125 | 0.98114 | 0.999656 |
| 17 | | 16 | 0.998281 | 0.998261 | 0.999991 |
| 18 | | 683 | 0.980902 | 0.980845 | 0.99974 |
| 19 | | 520 | 0.982179 | 0.982211 | 0.999861 |
| 20 | | 636 | 0.978626 | 0.978633 | 0.999658 |
| 21 | | 760 | 0.970704 | 0.971374 | 0.998923 |
| 22 | | 404 | 0.987293 | 0.987418 | 0.999702 |
| 23 | | 226 | 0.992395 | 0.992459 | 0.999962 |
| 24 | | 374 | 0.98082 | 0.981012 | 0.999508 |
| 25 | | 423 | 0.984275 | 0.984346 | 0.999905 |
| 26 | | 60 | 0.994059 | 0.993943 | 0.999984 |
| 27 | | 360 | 0.987792 | 0.987818 | 0.999882 |
| 28 | | 251 | 0.986966 | 0.987012 | 0.999917 |
| 29 | | 230 | 0.989675 | 0.989667 | 0.999956 |

**Table S2. Correlation between MI, RMI and r^2^ values on each chromosome (Human)**

| Chromosome | Number of SNP | RMI vs $r^{2}$ | MI vs$r^{2}$ | RMI vs MI |
| --- | --- | --- | --- | --- |
| 1 | 19682 | 0.938037 | 0.950346 | 0.97919 |
| 2 | 19016 | 0.940689 | 0.95266 | 0.979638 |
| 3 | 16819 | 0.938848 | 0.952761 | 0.977542 |
| 4 | 15781 | 0.941974 | 0.955551 | 0.978167 |
| 5 | 14470 | 0.942303 | 0.955261 | 0.978831 |
| 6 | 14565 | 0.939131 | 0.953692 | 0.976768 |
| 7 | 14095 | 0.939684 | 0.953352 | 0.977716 |
| 8 | 12357 | 0.94353 | 0.954179 | 0.981107 |
| 9 | 11601 | 0.941402 | 0.95391 | 0.978995 |
| 10 | 12718 | 0.939385 | 0.953351 | 0.977732 |
| 11 | 11465 | 0.940017 | 0.952896 | 0.979036 |
| 12 | 12649 | 0.940366 | 0.95372 | 0.97851 |
| 13 | 8978 | 0.941801 | 0.956368 | 0.977567 |
| 14 | 8551 | 0.943258 | 0.955832 | 0.979512 |
| 15 | 8215 | 0.945068 | 0.955494 | 0.982032 |
| 16 | 9197 | 0.940439 | 0.953024 | 0.979137 |
| 17 | 8953 | 0.944296 | 0.954603 | 0.981755 |
| 18 | 8153 | 0.94033 | 0.953615 | 0.978682 |
| 19 | 7753 | 0.944468 | 0.956215 | 0.980722 |
| 20 | 6656 | 0.945132 | 0.957137 | 0.9802 |
| 21 | 4053 | 0.9512 | 0.960128 | 0.984393 |
| 22 | 4759 | 0.949844 | 0.95854 | 0.984162 |

**Table S3. Correlation between MI, RMI and r^2^ values on each chromosome (Pig)**

| Chromosome | Number of SNP | RMI vs $r^{2}$ | MI vs$r^{2}$ | RMI vs MI |
| --- | --- | --- | --- | --- |
| 1 | 4888 | 0.959109 | 0.959007 | 0.999942 |
| 2 | 2787 | 0.964844 | 0.964827 | 0.999946 |
| 3 | 2460 | 0.960301 | 0.960147 | 0.99993 |
| 4 | 2788 | 0.964815 | 0.964772 | 0.99995 |
| 5 | 2119 | 0.975524 | 0.975495 | 0.999953 |
| 6 | 3034 | 0.97484 | 0.974918 | 0.999942 |
| 7 | 2173 | 0.96267 | 0.962438 | 0.999936 |
| 8 | 2974 | 0.961046 | 0.960923 | 0.999951 |
| 9 | 2794 | 0.960245 | 0.960164 | 0.999945 |
| 10 | 1806 | 0.961403 | 0.96146 | 0.999879 |
| 11 | 1764 | 0.96384 | 0.963818 | 0.999949 |
| 12 | 1305 | 0.957128 | 0.957045 | 0.999942 |
| 13 | 4112 | 0.966542 | 0.966419 | 0.999953 |
| 14 | 2885 | 0.969171 | 0.969105 | 0.999948 |
| 15 | 2359 | 0.971204 | 0.971151 | 0.999964 |
| 16 | 1681 | 0.965373 | 0.965284 | 0.999947 |
| 17 | 1447 | 0.955374 | 0.9553 | 0.999928 |
| 18 | 1122 | 0.958412 | 0.958368 | 0.999946 |

**Table S4. Correlation between MI, RMI and r^2^ values on each chromosome (Maize)**

| Chromosome | Number of SNP | RMI vs $r^{2}$ | MI vs$r^{2}$ | RMI vs MI |
| --- | --- | --- | --- | --- |
| 1 | 6823 | 0.955358 | 0.943358 | 0.970103 |
| 2 | 5052 | 0.972665 | 0.966322 | 0.985607 |
| 3 | 4865 | 0.980472 | 0.976583 | 0.990284 |
| 4 | 4745 | 0.957995 | 0.940357 | 0.979538 |
| 5 | 4675 | 0.95309 | 0.927285 | 0.975192 |
| 6 | 3466 | 0.970832 | 0.962148 | 0.9858 |
| 7 | 3605 | 0.968422 | 0.964565 | 0.991516 |
| 8 | 3675 | 0.978899 | 0.976428 | 0.993694 |
| 9 | 3149 | 0.967312 | 0.956424 | 0.98742 |
| 10 | 3035 | 0.969841 | 0.959294 | 0.988481 |

**Fig. S1. Main diagonal, histogram and fitted curves of LD value distributions for Human, Pig and Maize data sets.**


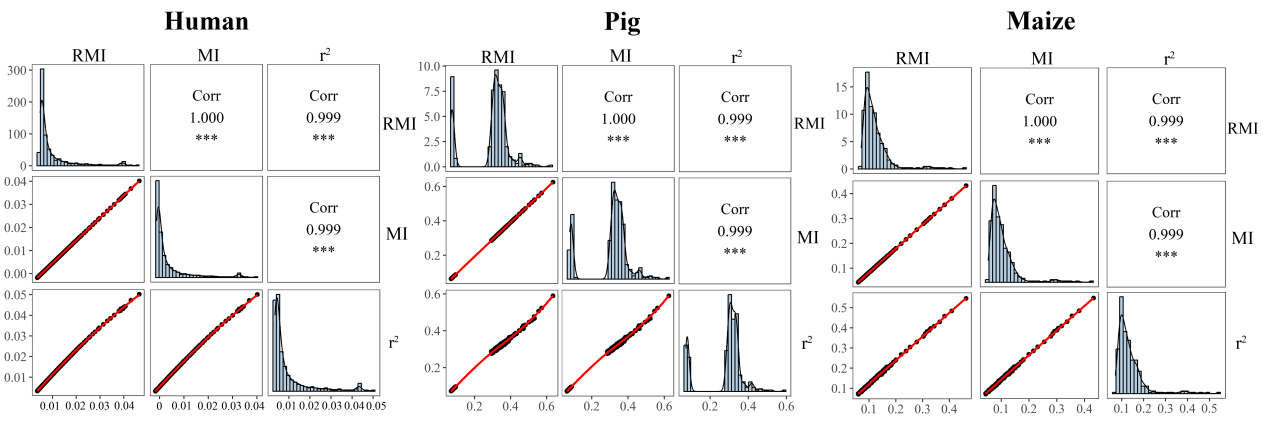

Supplement: jkad154_Supplementary_Data [file jkad154_supplementary_data.zip › Supplemental_Tables_and_Figures_G3-2023-404081.docx]
